# NeemAzal^®^-T/S Can Trigger Early Defense Responses in Susceptible Sunflower Seedlings Inoculated with *Plasmopara halstedii*: An Approach Based on the Enzymatic ROS Scavenging System

**DOI:** 10.3390/plants14223481

**Published:** 2025-11-14

**Authors:** Kevein Ruas Oliveira, Katalin Körösi, Balazs Barna, Rita Bán, Sarita Jane Bennett, Priscila Lupino Gratão

**Affiliations:** 1 Department of Integrated Plant Protection, Plant Protection Institute, Hungarian University of Agriculture and Life Sciences (MATE), 2100 Gödöllő, Hungary; kevein.ruas@gmail.com (K.R.O.); korosi.katalin.orsolya@uni-mate.hu (K.K.); barna.balazs@atk.hu (B.B.); ban.rita@uni-mate.hu (R.B.); 2 Department of Biology, School of Agricultural and Veterinary Sciences, São Paulo State University (UNESP), Jaboticabal 14884-900, Brazil; 3 Centre for Agricultural Research (HUN-REN CAR), Plant Protection Institute (NÖVI), Hungarian Academy of Sciences, 1116 Budapest, Hungary; 4 Centre for Crop and Disease Management, School of Molecular and Life Sciences, Curtin University, Perth, WA 6845, Australia; sarita.bennett@curtin.edu.au

**Keywords:** biotic stress, *Plasmopara halstedii*, biothrophs, oxidative stress, antioxidant responses, induced systemic resistance

## Abstract

Downy mildew, caused by *Plasmopara halstedii*, is a major threat to sunflower production worldwide, leading to severe yield losses. Since resistance in sunflower hybrids can be easily broken by the pathogen, it is important to find alternative and sustainable control methods against this disease. This study investigated the potential use of NeemAzal^®^-T/S (a neem-based biopesticide formulation) to induce antioxidant defense responses in sunflower seedlings inoculated with *P. halstedii* (pathotype 704). Its effects, alone, or in combination with a reduced dose of Mefenoxam, were evaluated under controlled conditions. Plant height, sporulation, antioxidant enzyme activities (SOD, CAT, APX, POX, and PPO), lipid peroxidation (MDA), and hydrogen peroxide (H_2_O_2_) contents were measured. Our results indicate that the antioxidant responses of seedlings varied according to the treatment. MDA levels decreased even when NeemAzal^®^-T/S was applied alone, while H_2_O_2_ production only decreased when both treatments were applied combined. Overall, NeemAzal^®^-T/S can be a valuable alternative strategy to help control sunflower downy mildew, since it reduced sporulation and MDA content, and increased APX, POX, and PPO activities even at a later stage of infection in susceptible seedlings. These findings indicate that NeemAzal^®^-T/S can activate defense mechanisms associated with oxidative stress reduction in sunflower, offering a promising strategy to help manage downy mildew in a more sustainable manner.

## 1. Introduction

Sunflower (*Helianthus annuus* L.) is one of the most important oilseed crops in the world, grown worldwide in temperate, sub-tropical and tropical climates under a wide range of environments [1]. It is also one of the top five oilseed crops globally, with major production regions in the European Union, Russia, Ukraine, Argentina, and the USA during the 2024/2025 season [2,3].

Downy mildew is a major disease caused by the biotrophic oomycete (*Plasmopara halstedii* (Farl) Berlese et de Toni). This pathogen can cause yield losses of greater than 85% in sunflower [4], with current control methods relying on the use of resistant cultivars, cultural methods, and fungicides. Seeds are usually coated and treated with fungicides. However, due to the high phenotypic diversity of the pathogen, regarding its virulence and fungicide sensitivity [5], effective disease control requires a broader understanding of its biology, physiological capabilities and requirements, as well as the molecular mechanisms underlying its interactions with both the host and the environment [6]. Resistance breeding can be easily broken as the pathogen can rapidly develop different pathotypes developing fungicide resistance and overcoming plant resistance genes (P1 resistance genes) [4,7]. As a result, crop protection costs against this pathogen can be very high. Therefore, alternate solutions which are sustainable, economical, and eco-friendly should be used to control this disease [8].

The use of plant-derived compounds has been increasing worldwide in agricultural systems as plant protection products. Botanical compounds derived from the neem tree (*Azadirachta indica* A. Juss.) have been used for their role in sustainable pest management. Among these, azadirachtin is recognized as the main bioactive limonoid responsible for most of neem’s insecticidal properties, being commonly used in integrated pest management (IPM) systems due to its low toxicity to non-target organisms and environmental safety. This tetranortriterpenoid compound acts as an insect growth regulator, antifeedant, and repellent [9]. Beyond its insecticidal activity, different studies have demonstrated that azadirachtin and other neem-derived limonoids, such as nimbin and salannin, also exhibit antifungal effects [10,11,12]. These compounds have been shown to inhibit sporulation and mycelial growth of several plant pathogens, including *P. halstedii*, the causal agent of sunflower downy mildew [7,13]. However, most of these studies have been focused on the direct antifungal properties of neem-based formulations, rather than their potential to induce plant resistance [14].

NeemAzal^®^-T/S is a neem-based botanical formulation which contains azadirachtin A as the main active compound, along with other limonoids that may also exhibit biological activity against insects and plant pathogens [7,9]. Although it is primarily registered in Europe as a biological pesticide [15], some studies have demonstrated its potential role in suppressing pathogens and inducing plant defense response [7,13,14]. For instance, different studies have demonstrated its antifungal properties as being of fungitoxic phenolic compounds [10,11,12]. However, these studies do not elucidate clearly if it is capable of inducing resistance in plants through increased enzymatic activity in the ROS scavenging system. Even though NeemAzal^®^-T/S is not currently approved for use in disease management in Europe [15], its components have been widely studied for their biochemical effects on plant defense pathways, since they possess antifeedant, antifungal, nematocidal, and insecticidal properties [9]. Therefore, evaluating the biochemical and physiological effects of a neem-based botanical formulation on sunflower seedlings inoculated with *P. halstedii* can provide valuable insight into its potential use as a botanical inducer of resistance in addition to its known biopesticide effects [14].

Plant defense against pathogens is regulated by a complex network of different signals [16]. A common and early response of plants to both abiotic and biotic stresses is an oxidative burst, which is the rapid accumulation of reactive oxygen species (ROS) [16,17,18,19]. To mitigate the harmful effects of ROS, plants activate a defense system involving the production of both enzymatic and non-enzymatic antioxidants [20,21,22]. While ROS play a crucial role in defense signaling and restricting pathogen invasion, excessive ROS accumulation can lead to oxidative damage and programmed cell death. In addition to their role in regulating plant growth and development, ROS are central to the oxidative burst that initiates defense processes such as pathogen restriction, cell death, and signal transduction [18]. Enhancing antioxidant enzyme activity enables plants to better tolerate ROS-induced cellular damage [23,24]. However, some pathogens can manipulate plant defense signaling to their advantage by altering phytohormone balances, which disrupts normal defense responses and triggers maladaptive activation of antioxidant pathways [18,25].

Previous studies have demonstrated that NeemAzal^®^-T/S could effectively help to control sunflower downy mildew [7,13]. However, the mechanisms which result in its induced systemic resistance (ISR) are still poorly understood. It is important to note that in the sunflower and downy mildew plant-pathogen interaction, it has been demonstrated that benzothiadiazole (BTH) significantly reduced disease symptoms and enhanced the activity of some defense-related enzyme in susceptible sunflower plants [22]. In this way, it is hypothesized that the use of NeemAzal^®^-T/S could increase resistance to downy mildew in susceptible sunflower seedlings through an intensified antioxidant plant defense system which could result in a better regulation of ROS production and degradation. Therefore, we aimed to characterize defense responses taking place in sunflower plants inoculated with *P. halstedii* and treated with NeemAzal^®^-T/S by measuring the activities of major antioxidant enzymes, lipid peroxidation (MDA) and hydrogen peroxide (H_2_O_2_) content, as well as disease severity (sporulation), increasing the knowledge on how these impacts could affect seedling development or leads to plant death.

## 2. Materials and Methods

### 2.1. Plant Materials and Growth Conditions

The experiment was carried out at the Plant Protection Institute, Department of Integrated Plant Protection, Hungarian University of Agriculture and Life Sciences (MATE), Gödöllő, Hungary. The susceptible Hungarian open pollinated sunflower cultivar (Iregi Szürke Csíkos) was used, as it is highly susceptible to all known pathotypes of downy mildew. Sunflower seedlings were grown in pots filled with perlite, placed in a growth chamber under controlled conditions (22 °C, photoperiod of 12 h, and 60% relative humidity of the air) and watered as required.

### 2.2. Pathogen Inoculation and Experimental Conditions

The *P. halstedii* pathotype 704 from the Hungarian University of Agriculture and Life Sciences collection was used to obtain a compatible plant-pathogen interaction [26]. Downy mildew infection in sunflower seedlings is strongly influenced by environmental conditions, particularly temperature and humidity. In our study, all experiments were conducted in a growth chamber under strictly controlled conditions to ensure that disease development was solely due to *P. halstedii* (pathotype 704). Temperature and relative humidity were maintained at levels optimal for pathogen germination and infection, while a nutrient solution was carefully and regularly provided to support healthy seedling growth and pathogen activity. Only the specified pathogen was present, and all plants were grown in sterile substrate (perlite), preventing infections by other pathogens. These controlled conditions allowed accurate evaluation of disease incidence, symptom development, and treatment effects, ensuring that observed differences are attributable exclusively to the inoculated pathogen and applied treatments.

### 2.3. Preparation of NeemAzal^®^-T/S (AZA) and Mefenoxam (MEF)

NeemAzal^®^-T/S (AZA) was used to induce resistance against *P. halstedii* (pathotype 704). A working concentration (0.1%) was prepared of AZA obtained from Trifolio Gmbh, Germany, containing (1% azadirachtin A), a registered plant protection commercial product in the European Union, by dissolving 1 mL of AZA in 100 mL of solution. Mefenoxam (MEF) (Apron XL 350 FS—350 g/L Mefenoxam, Syngenta AG, Basel, Switzerland) was dissolved as per the EU registered rate (3 mg/kg seeds), and half of its dose (1.5 mg/kg seeds), depending on the treatment, by homogeneously coating the seeds and keeping them at room temperature until they were completely dry [7,13].

### 2.4. Treatments and Pathogen Inoculation

Sunflower seeds of the susceptible Hungarian cultivar Iregi Szürke Csíkos were first sterilized in a 1% Na-hypochlorite solution for 3–5 min, rinsed thoroughly with running tap water and germinated on moist filter paper at room temperature until 3–5 mm long root initials had developed (2–3 days). The Whole Seedling Immersion (WSI) method was used for this experiment [27]. Three-day-old germinated seedlings (25 seedlings) were then firstly immersed in NeemAzal^®^-T/S (AZA) containing 0.1% azadirachtin A as the main active compound for 2 h. These pre-treated seedlings were subsequentially inoculated with the *P. halstedii* pathotype 704 by immersing them for 5 h in a sporangial suspension (50,000 sporangia/mL), and then incubating them at 16 °C overnight in the dark [27]. For negative control plants, healthy, untreated and non-inoculated seedlings were immersed in bidistilled water (BW) for 2 h. For positive controls, seedlings were first treated with mefenoxam: INO + MEF treatment (standard dose) or INO + MEF + AZA (50% of the standard MEF dose). Seedlings were then transplanted to pots filled with perlite (5 seedlings per pot), with ten replicates, and placed in a growth chamber under controlled conditions (22 °C, photoperiod of 12 h, and 60% relative humidity), and watered as required with a nutrient solution supplemented with the nutrients (*m*/*m*%): nitrogen (N) 14%, phosphorus pentoxide (P_2_O_5_) 7%, potassium oxide (K_2_O) 21%, magnesium oxide (MgO) 1%, boron (B) 1%, calcium (Ca) 1%, iron (Fe) 1%, manganese (Mn) 1% and zinc (Zn) 1%. The nutrient solution was replaced once per week and its pH adjusted to 6.0 ± 0.5 using either sodium hydroxide (NaOH) or hydrochloric acid (HCl) solutions. Treatments were as following:

- Seedlings treated with bidistilled water (CONTROL).
- Seedlings inoculated with *P. halstedii* sporangial suspension (INO).
- Seedlings pre-treated with MEF (standard dose of 3 mg/kg seeds) and inoculated with *P. halstedii* sporangial suspension (INO + MEF).
- Seedlings pre-treated with NeemAzal^®^-T/S (AZA) and inoculated with *P. halstedii* sporangial suspension (INO + AZA).
- Seedlings pre-treated with NeemAzal^®^-T/S (AZA) + MEF (1.5 mg/kg seeds—50% of the standard dose) and inoculated with *P. halstedii* sporangial suspension (INO + AZA + MEF).

### 2.5. Assessing Disease Incidence

Ten days after inoculation, when the first true leaves (1 mm in length) had developed, bidistilled water was sprayed on all plants using a hand sprayer. All pots were kept overnight in completely darkness at 19 °C to induce sporulation on enclosed trays with a lid, covered in a dark polyethylene bag (to saturate it with moisture). The first evaluation was performed at 09 dpi, based on cotyledons bearing sporangia (white growth). Disease incidence was made by using a 0−1 scale (cotyledon leaf bearing sporangia or not): no sporulation (0), and sporulating (1) [13,22]. Plant growth (plant height) was also measured. For the second evaluation (presence or absence of chlorosis and seedling damping-off) 20 days post inoculation (dpi), plants were returned to the growth chamber under controlled conditions (22 °C, photoperiod of 12 h and 60% relative humidity), and watered as required with the nutrient solution.

### 2.6. Lipid Peroxidation (MDA) and Hydrogen Peroxide (H_2_O_2_) Content

Plant tissue (0.5 g of seedling hypocotyl) was ground with 20% (*w*/*v*) polyvinylpyrrolidone (PVPP) and 0.1% trichloroacetic acid (TCA). Following centrifugation at 11,000× *g* for 10 min, the supernatant was mixed with a solution of 20% TCA and 5% thiobarbituric acid (TBA) and incubated in a water bath at 95 °C for 30 min. The reaction was stopped by cooling in an ice bath for 10 min, followed by another centrifugation at 11,000× *g* for 10 min [28]. Lipid peroxidation was estimated by measuring the thiobarbituric acid reactive substances (TBARS) content. The concentration of malondialdehyde (MDA) equivalents was calculated using an extinction coefficient of 1.55 × 10^5^ mol^−1^ cm^−1^, with absorbance readings taken at 535 and 600 nm [28]. Results were expressed as μmol MDA/mg fresh tissue.

H_2_O_2_ content was determined as following: plant tissue (0.5 g of seedling hypocotyl) was homogenized in 0.1% thiobarbituric acid and centrifuged at 10,000× *g* for 10 min. The supernatant was collected, and 100 mM potassium phosphate buffer (pH 7.5) along with 1 M potassium iodide solution were added. The mixture was then incubated on ice for 1 h. H_2_O_2_ content was quantified by comparing the absorbance at 390 nm to a standard curve generated with a known concentration of H_2_O_2_ [29].

### 2.7. Determination of Total Soluble Proteins

Plant extracts were used to evaluate the activities of antioxidant enzymes in sunflower seedlings subjected or not to inoculation with *P. hasteldii* and treated or not NeemAzal^®^-T/S (AZA) or Mefenoxam (MEF). For that, plant tissue (0.5 g of hypocotyl) was ground and homogenized in a cold mortar pestle (4 °C) with 20% (*w*/*v*) polyvynilpyrrolidone (PVPP) and 3 mL of TRIS-HCl buffer (50 mM, pH 7.8), containing 1 mM EDTA-Na_2_ and 7.5% (*w*/*v*) PVPP (Sigma-Aldrich Co., St. Louis, MO, USA), plus sterile sand to increase friction. Samples were then centrifuged at 10,000× *g* for 20 min at 4 °C [23]. Total soluble protein content in sunflower seedlings was measured using bovine serum albumin serving as the standard [30]. Plant tissue (0.5 g of hypocotyl) was harvested and homogenized in a buffer at a ratio of 2:1 (buffer volume to fresh weight) using a mortar and pestle. The buffer consisted of 100 mM potassium phosphate (pH 7.5), 1 mM EDTA, 3 mM DL-dithiothreitol, and 5% (*w*/*v*) insoluble polyvinylpolypyrrolidone [31]. The homogenate was centrifuged at 10,000× *g* for 30 min, and the supernatant (plant extract) was then kept on melting ice and used for subsequent enzymatic assays.

### 2.8. Antioxidant Enzymes

Enzyme activity was determined spectrophotometrically using a SmartSpec Plus Spectrophotometer (BioRad, Hercules, CA, USA) at 25 °C. Samples for enzyme assays were collected at 3-, 9- and 21-day-old plants inoculated with *P. halstedii* [22]. Superoxide dismutase (SOD, EC 1.15.1.1), catalase (CAT, EC 1.11.1.6), ascorbate peroxidase (APX, EC 1.11.1.11), peroxidase (POX, EC 1.11.1.7) and polyphenol oxidase (PPO, EC 1.10. 3.1) enzyme activities were determined:

SOD activity was measured as following: the reaction was carried out in a reaction chamber (box) illuminated with 15 W fluorescent lightbulbs at 25 °C. A 50 μL aliquot of the sample was added to a 5 mL reaction mixture containing sodium phosphate buffer (50 mmol L^−1^, pH 7.8), methionine (13 mmol L^−1^), NBT (75 mmol L^−1^), EDTA (0.1 mmol L^−1^), and riboflavin (2 μmol L^−1^). The tubes were placed inside the closed chamber, shielded from external light, and exposed to the artificial light for 15 min to facilitate the formation of blue formazan due to the photoreaction of nitroblue tetrazolium (NBT). Parallel samples, prepared identically but wrapped in aluminum foil to prevent light exposure, were used as controls. After 15 min, the contents were vortexed to homogenize the mixture. Absorbance readings were taken at 560 nm using a spectrophotometer, and the SOD activity was expressed as U SOD mg^−1^ protein [32].

CAT activity was measured spectrophotometrically at 25 °C using a reaction mixture consisting of 1 mL of 100 mM potassium phosphate buffer (pH 7.5) and 25 mL of a 30% H_2_O_2_ solution. Enzyme activity was determined by tracking the decrease in H_2_O_2_ absorbance at 240 nm over a 1-min period. CAT activity was reported as µmol min^−1^ mg^−1^ protein [33].

APX activity was determined spectrophotometrically using a reaction mixture containing the seedling extract, 80 mM potassium phosphate buffer (pH 7.0), 5 mM ascorbate, 1 mM EDTA, and 1 mM H_2_O_2_. The activity was assessed by measuring the rate of ascorbate oxidation at 290 nm at 30 °C. APX activity was expressed as µmol ascorbate min^−1^ mg^−1^ protein [28].

POX activity was determined as follows: the reaction mixture contained 2.2 mL potassium-phosphate buffer (0.1 M, pH 6), 100 μL of 50 mM guaiacol, 100 μL of 12 mM H_2_O_2_, and 100 μL plant extract. Spectrophotometer measurements lasted 100 s at 470 nm. POX activity was calculated using the extinction coefficient of the formed product (26.6 mM^−1^cm^−1^) and it was expressed as μM H_2_O_2_ × g^−1^ fresh weight × min^−1^ [34].

PPO activity was determined by measuring the rate of quinone formation, as indicated by an increase in absorbance at 400 nm. The reaction mixture contained 2.2 mL potassium-phosphate buffer (0.1 M, pH 6.0), 1 mM EDTA-Na_2_, 20 mM catechol and 200 μL plant extract. Spectrophotometer measurements lasted 100 s and PPO activity was calculated using the extinction coefficient of the formed product (950 M^−1^cm^−1^), being expressed as μM catechol × g^−1^ fresh weight × min^−1^ [22].

### 2.9. Statistical Analyses

The experiment was carried out as a completely randomized design in a factorial scheme (3 × 5): three different times for enzymes measurements and five treatments: CONTROL, INO, INO + MEF, INO + AZA and INO + AZA + MEF. Results were expressed as the mean and standard error of the mean (SEM) of three independent replicates for each plant extract for enzymes, H_2_O_2_ and MDA (15 seedlings, 3 pots containing 5 plants). For plant growth (plant height) and sporulation, results were expressed as the mean and standard error of the mean (SEM) of ten independent replicates (50 seedlings, 10 pots containing 5 plants). Statistical analysis was performed using the software AgroStat (v. 1.1.0.694) [35]. The obtained data were subjected to analysis of variance (ANOVA), and when significant, means were compared using Tukey’s post hoc test at 5% probability (*p* < 0.05). The experimental design, including both negative (non-inoculated, untreated) and positive (INO + MEF) controls, and appropriate replication, ensures that observed differences in disease incidence, oxidative stress, and antioxidant enzymes activities can be confidently attributed to the applied treatments under controlled conditions.

## 3. Results

**Figure 1:**
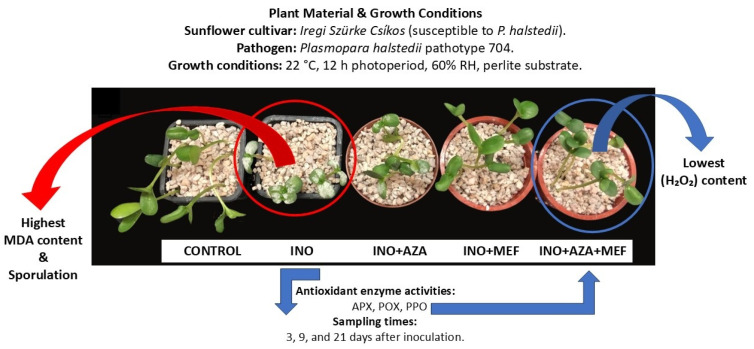
Schematic representation of the main findings of the experiment showing treatments, pathogen inoculation, growth conditions, and biochemical analyses performed on sunflower seedlings. Susceptible sunflower seedlings (Iregi Szürke Csíkos) were inoculated with downy mildew (pathotype 704) and treated with NeemAzal^®^-T/S (AZA), Mefenoxam (MEF—standard dose) and AZA + MEF (50% of the standard MEF dose). CONTROL (non-inoculated and non-treated seedlings), INO (inoculated and non-treated seedlings), INO + AZA (inoculated and AZA-treated seedlings), INO + MEF (inoculated and MEF–treated seedlings, standard dose), and INO + AZA + MEF (inoculated and AZA + MEF-treated seedlings, 50% of the standard MEF dose).

The variables evaluated in this study—disease incidence (sporulation), seedling growth (plant height), antioxidant enzymes and the oxidative stress (MDA and H_2_O_2_)—all showed significant responses. Analysis of treatment effects indicates clear interactions between pathogen inoculation and plant protection applications. While NeemAzal^®^-T/S (AZA) or Mefenoxam (MEF) alone partially reduced disease incidence and oxidative stress markers, the combined treatment (INO + AZA + MEF) consistently resulted in synergistic effects (Figure 1), leading to the lowest H_2_O_2_ accumulation, reduced MDA content, enhanced activity of antioxidant enzymes (APX, POX, and PPO), and increased plant height across different measuring times (days post inoculation).

### 3.1. Disease Incidence

**Figure 2:**
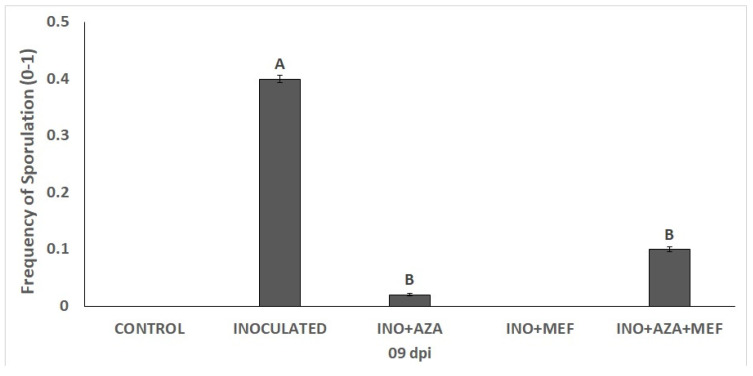
Frequency of sporulation in susceptible sunflower seedlings inoculated with downy mildew (pathotype 704) and treated with NeemAzal^®^-T/S (AZA), Mefenoxam (MEF—standard dose) and AZA + MEF (50% of the standard MEF dose). Treatments are as follows: INO (inoculated and non-treated seedlings), INO + AZA (inoculated and AZA-treated seedlings), and INO + AZA + MEF (inoculated and AZA + MEF-treated seedlings, 50% of the standard MEF dose). CONTROL (non-inoculated, non-treated seedlings) and INO + MEF (inoculated seedlings treated with the standard MEF dose) showed zero sporulation and are not shown in the figure. Error bars represent ±SEM of ten replicates. Different letters above columns indicate significant differences between treatments at *p* < 0.05 following Tukey’s post hoc test.

For disease incidence, the frequency of sporulation significantly varied among the different treatments. As expected, it was observed that sporulation occurred more severely in seedlings of the inoculated treatment (INO), where there was no application of any plant protection product, indicating severe disease development. In contrast, treatments involving AZA applications showed a marked reduction in sporulation. The use of fungicide, more specifically in the INO + MEF treatment, resulted in zero sporulation frequency, demonstrating effective disease suppression. Similarly, the INO + AZA + MEF treatment reduced sporulation to intermediate levels, being significantly lower than the INO treatment and statistically equal to the INO + AZA treatment (Figure 2).

The INO+MEF treatment completely suppressed sporulation, being comparable to the non-inoculated CONTROL treatment. The same was not observed for seedlings only treated with AZA or the combination of MEF+AZA, as in, these treatments plants still showed some degree of sporulation, besides being a lower frequency than in the INO treatment (Figure 2).

### 3.2. Seedling Growth (Initial and Final Plant Height)

**Figure 3:**
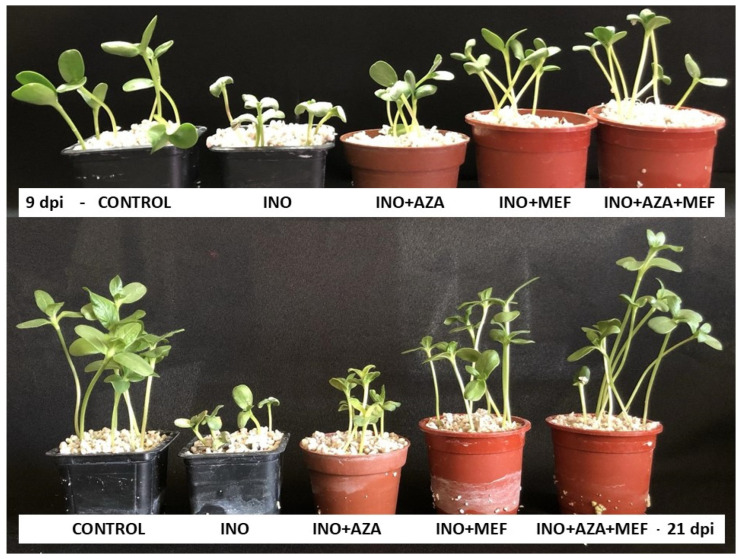
Initial (9 dpi) and final (21 dpi) plant height in susceptible sunflower seedlings inoculated and non-inoculated with *P. halstedii* (pathotype 704) and treated with NeemAzal^®^-T/S (AZA), Mefenoxam (MEF—standard dose) and AZA + MEF (50% of the standard MEF dose). CONTROL (non-inoculated and non-treated seedlings), INO (inoculated and non-treated seedlings), INO + AZA (inoculated and AZA-treated seedlings), INO + MEF (inoculated and MEF–treated seedlings, standard dose), and INO + AZA + MEF (inoculated and AZA + MEF-treated seedlings, 50% of the standard MEF dose).

For seedling growth, plant height was significantly affected by both pathogen infection and treatment applications. Seedlings from the INO treatment showed a marked reduction in both initial (9 dpi) and final (21 dpi) plant heights, which were the lowest among all treatments. This clearly indicates the negative impact of *P. halstedii* infection on seedling growth. In contrast, the non-inoculated CONTROL treatment achieved the highest final plant height, indicating healthy growth under disease-free conditions (Figure 3).

**Figure 4:**
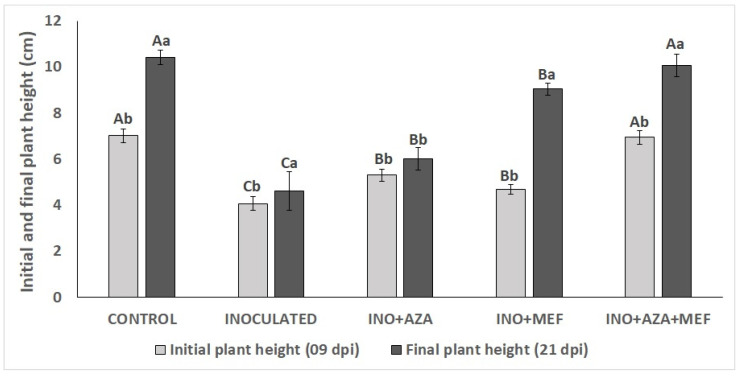
Initial (9 dpi) and final (21 dpi) plant height in susceptible sunflower seedlings inoculated and non-inoculated with *P. halstedii* (pathotype 704) and treated with NeemAzal^®^-T/S (AZA), Mefenoxam (MEF—standard dose) and AZA + MEF (50% of the standard MEF dose). CONTROL (non-inoculated and non-treated seedlings), INO (inoculated and non-treated seedlings), INO + AZA (inoculated and AZA-treated seedlings), INO + MEF (inoculated and MEF–treated seedlings, standard dose), and INO + AZA + MEF (inoculated and AZA + MEF-treated seedlings, 50% of the standard MEF dose). Error bars represent ±SEM of ten replicates. Different uppercase letters above columns indicate significant differences between treatments, while lowercase letters indicate significant differences between initial and final plant height within a treatment at *p* < 0.05 following Tukey’s post hoc test.

Significant effects of AZA and MEF applications on plants and between treatments were observed. It can be seen that the treatments (INO + AZA and INO + MEF) showed the second highest initial seedling growth. However, only the seedlings inoculated with the pathogen and treated with both combined (INO + AZA + MEF) showed initial seedling growth equal to the CONTROL treatment. Notably, the combination treatment INO + AZA + MEF restored plant height to levels statistically similar to the non-inoculated CONTROL treatment (Figure 4).

### 3.3. Lipid Peroxidation and H_2_O_2_ Content

**Figure 5:**
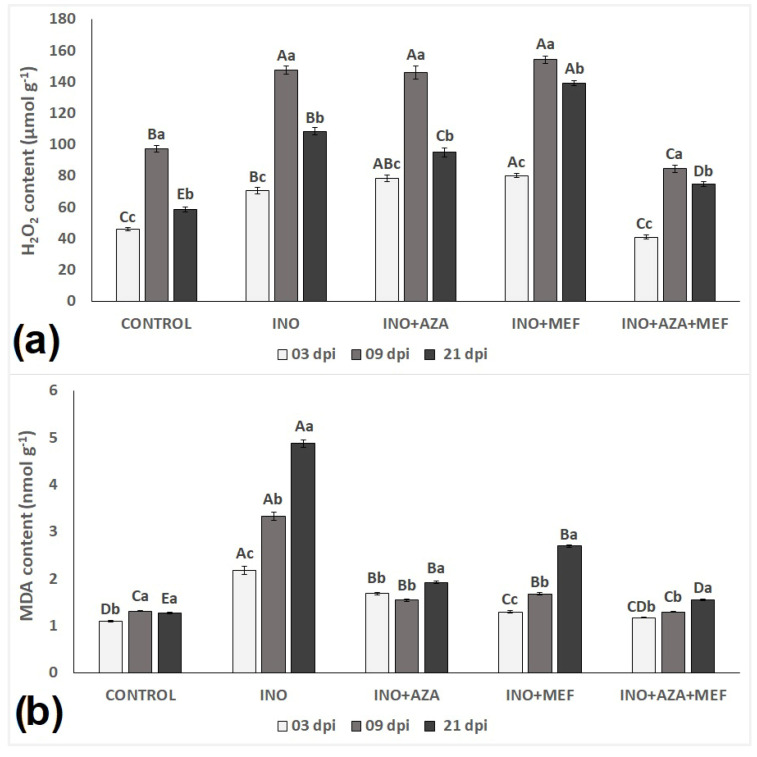
Lipid peroxidation (**a**), measured as malondialdehyde (MDA) (nmol g^−1^) and hydrogen peroxide content (**b**) (H_2_O_2_) (µmol g^−1^) in sunflower seedlings inoculated and non-inoculated with downy mildew (pathotype 704) and treated with NeemAzal^®^-T/S (AZA), Mefenoxam (MEF—standard dose) and AZA + MEF (50% of the standard MEF dose). CONTROL (non-inoculated and non-treated seedlings), INO (inoculated and non-treated seedlings), INO + AZA (inoculated and AZA-treated seedlings), INO + MEF (inoculated and MEF–treated seedlings, standard dose), and INO + AZA + MEF (inoculated and AZA + MEF-treated seedlings, 50% of the standard MEF dose). Error bars represent ±SEM of three replicates. Different uppercase letters above columns indicate significant differences between treatments, while lowercase letters indicate significant differences between 3, 9 and 21 dpi at *p* < 0.05 following Tukey’s post hoc test.

Malondialdehyde (MDA) content, an indicator of lipid peroxidation and oxidative stress, exhibited significant variation across treatments and time points (Figure 5b). Seedlings of the INO treatment naturally showed a high MDA content when compared to all other treatments. In fact, MDA content in inoculated and non-treated seedlings progressively increased over time, being the highest amongst all treatments at 3, 9 and 21 dpi, indicating severe oxidative damage due to pathogen infection. The lowest MDA content, as expected, was found in CONTROL plants, and it did not vary much regardless of plant age, being the same at 9 and 21 dpi, reflecting minimal oxidative stress. (Figure 5a).

Both treatments AZA or MEF alone seemed to have had some effect in reducing MDA content in inoculated seedlings in all evaluations, when compared to only inoculated and non-treated plants (INO treatment). The INO + AZA treatment effectively limited MDA accumulation, with only a slight increase in MDA content from 9 to 21 dpi. On the other hand, seedlings from the INO + MEF treatment, in their final evaluation (21 dpi), presented a high MDA content when compared to the CONTROL treatment (Figure 5a). The combined use of both, AZA + MEF had a positive effect in reducing MDA content and kept it low over time, as it did not vary much from 3 to 21 dpi. Seedlings from this treatment (INO + AZA + MEF) presented MDA content very similar to CONTROL plants, with only a slightly increase from 9 to 21 dpi (Figure 5a).

Hydrogen peroxide (H_2_O_2_), an indicator of reactive oxygen species (ROS) accumulation and oxidative stress signalling, was significantly influenced by both pathogen infection and treatment over time (Figure 5b). For H_2_O_2_ content, a different pattern, as opposed to the MDA content, was observed in seedlings and amongst treatments. Overall, in plants of all treatments, H_2_O_2_ content increased from 3 to 9 dpi, and then decreased from 9 to 21 dpi (Figure 5b). Interestingly, H_2_O_2_ content at 3 dpi was highest in the INO + AZA and INO + MEF treatments, being slightly higher than the INO treatment. Only seedlings from the INO + AZA + MEF treatment presented a low H_2_O_2_ content at 3 dpi which was comparable to the CONTROL treatment (Figure 5b).

No statistical difference was found between the INO, INO + AZA and INO + MEF treatments with seedlings of these treatments presenting the highest H_2_O_2_ content recorded at 9 dpi (Figure 5b). Surprisingly, plants of the INO + AZA + MEF treatment presented the lowest H_2_O_2_ content recorded at 9 dpi, even lower than values found in the CONTROL treatment. Overall, H_2_O_2_ content increased in all plants from 3 to 9 dpi, following a decrease in its content from 9 to 21 dpi. However, it is worth noting that this decrease was more pronounced in plants of the CONTROL and INO + AZA + MEF treatments (Figure 5b).

### 3.4. Antioxidant Enzymes in Healthy, Treated and/or Infected Sunflower Seedlings

**Figure 6:**
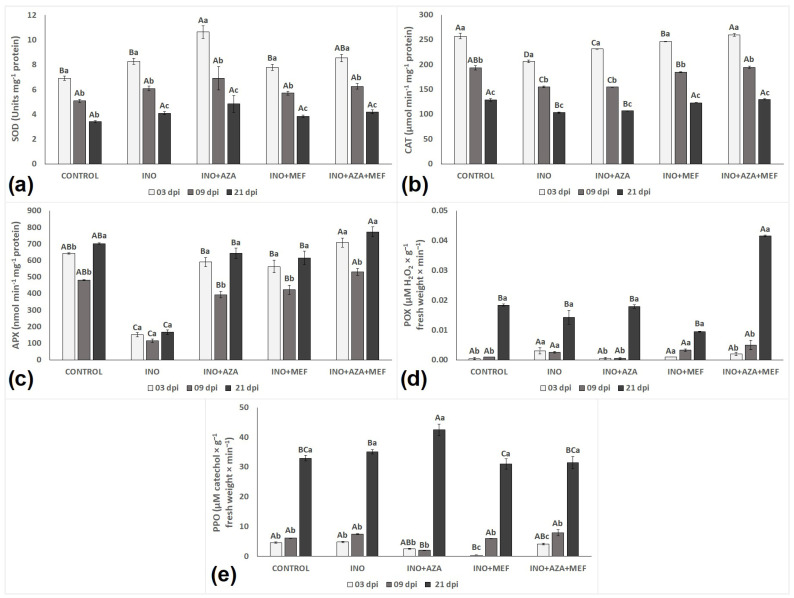
The activity of major antioxidant enzymes in sunflower seedlings inoculated or not with downy mildew (pathotype 704) and treated with NeemAzal^®^-T/S (AZA), Mefenoxam (MEF—standard dose) and AZA + MEF (50% of the standard MEF dose): SOD (**a**), CAT (**b**), POX (**c**), APX (**d**), and PPO (**e**). CONTROL (non-inoculated and non-treated seedlings), INO (inoculated and non-treated seedlings), INO + AZA (inoculated and AZA-treated seedlings), INO + MEF (inoculated and MEF–treated seedlings, standard dose), and INO + AZA + MEF (inoculated and AZA + MEF-treated seedlings, 50% of the standard MEF dose). Error bars represent ±SEM of three replicates. Different uppercase letters above columns indicate significant differences between treatments, while lowercase letters indicate significant differences between 3, 9 and 21 dpi at *p* < 0.05 following Tukey’s post hoc test.

Antioxidant responses in healthy (CONTROL), infected (INO) and inoculated-treated plants (INO + MEF, INO + AZA and INO + AZA + MEF) varied amongst treatments and over time (Figure 4). Superoxide dismutase (SOD), catalase (CAT) and ascorbate peroxidase (APX) showed a very different pattern than that observed for peroxidase (POX) and polyphenol oxidase (PPO) enzymes (Figure 6).

When it comes the antioxidant response of plants, SOD activity remained relatively stable in seedlings from the CONTROL treatment, with a slight decline over time, reflecting normal physiological processes. In contrast, inoculated untreated plants (INO treatment) showed an initial increase in SOD, followed by a significant decline from 9 dpi to 21 dpi, suggesting an early but not sustained defence response that deteriorated over time under pathogen stress. For this enzyme, the highest value recorded was found in seedlings from the INO + AZA treatment, at 3 dpi (Figure 6a). However, this elevated activity also declined at subsequent time points over time, even though it remained higher than the INO treatment (Figure 6a). A similar pattern was observed in the INO + AZA + MEF treatment, where SOD activity was highest at 3 dpi, indicating an early and robust activation of ROS-scavenging defences. This pattern suggests that the combined treatment might have contributed to an early and effective ROS detoxification response. Overall, SOD activity progressively decreased over time in seedlings of all treatments with the lowest values recorded at 21 dpi and no significant differences observed between treatments except the CONTROL in the last measurement (Figure 6a).

CAT activity followed a similar trend to that observed for SOD, with a similar pattern and a decrease in activity for seedlings of all treatments over time, with the lowest values recorded in plants at 21 dpi (Figure 6b). In the CONTROL plants, CAT activity was consistently high at early stages, but decreased progressively over time, reflecting normal plant development under non-stressed conditions. In the inoculated untreated plants (INO treatment), CAT activity was noticeably lower when compared to all other treatments, starting at 3 dpi, then significantly decreasing at 9 and 21 dpi, suggesting that pathogen infection might have compromised the antioxidative defence system. Both, INO + AZA and INO + MEF treatments effectively enhanced CAT activity in stages of infection (3 dpi), but this was followed by a decreased at 9 and 21 dpi, suggesting a strong but not maintained initial activation of the antioxidant system. Interestingly, the combined treatment (INO + AZA + MEF) maintained the highest CAT activity among inoculated plants, similar to the CONTROL treatment at an early stage of infection (3 dpi) and still relatively high at 9 dpi, before declining at 21 dpi (Figure 6b). This might indicate a synergistic effect of AZA and MEF in enhancing ROS-scavenging capacity.

APX activity showed a completely different pattern to that observed for SOD and CAT, with significant differences between treatments and over time (Figure 6c). Our results show that the lowest values recorded for APX activity happened to be in the INO treatment, while the highest APX activity was observed in seedlings of the CONTROL and INO + AZA + MEF treatments. In seedlings from the CONTROL treatment, APX activity was stable and high, slightly decreasing at 9 dpi, before increasing again at 21 dpi. This reflects the normal antioxidative balance in non-stressed plants. In contrast, the inoculated untreated seedlings (INO treatment) exhibited severely suppressed APX activity, and remained consistently low across all measurement times (3, 9 and 21 dpi) over time. This suggests a failure to activate the antioxidant defence under pathogen stress. Even though all inoculated and treated seedlings showed a high APX activity at an early stage of infection, the highest APX activity at 3 dpi was observed in seedlings from the INO + AZA + MEF treatment (Figure 6c). Interestingly, it was only when measuring APX that a decrease from 3 to 9 dpi was followed by an increase from 9 to 21 dpi in seedlings of all treatments. This pattern was not observed for any other enzyme measured in this study. Both treatments when applied in isolation (INO + AZA and INO + MEF) significantly increased APX activity when compared to the INO treatment at 3 dpi, with a slight decrease at 9 dpi followed by an increase again at 21 dpi. Notably, the combined treatment (INO + AZA + MEF) achieved the highest APX activity among inoculated plants, maintaining levels similar to or even exceeding the CONTROL treatment at 21 dpi (Figure 6c). This indicates a synergistic or additive effect of AZA to MEF in boosting antioxidative capacity, reflecting its role as a resistance inducer.

When quantifying POX, it was clear that its activity, as opposed to SOD, CAT and APX, increased in all treatments over time, with the highest activity for this enzyme observed in seedlings of the combined treatment (INO + AZA + MEF) at 21 dpi (Figure 6d). In seedlings from the CONTROL treatment, POX activity remained low at 3 and 9 dpi, but significantly increased at 21 dpi, suggesting a natural increase related to plant growth rather than a stress response. The inoculated untreated plants (INO treatment) showed a similar trend with low activity at 3 and 9 dpi, followed by a moderate increase at 21 dpi, indicating that infection alone did not induce POX activity over time. A similar pattern was also observed for the INO + AZA and INO + MEF treatments, where POX activity remained relatively similar to the INO treatment, indicating that infection under either treatment in isolation did not induce POX activity over time. The highest POX activity was observed in the INO + AZA + MEF combination at 21 dpi, which was significantly higher than in all other treatments (Figure 6d). This sharp increase suggests a synergistic activation of POX-related defence mechanisms when both AZA and MEF were applied together, indicating azadirachtin’s moderate role in POX activation.

Following a similar trend to POX, PPO activity did not increase from 3 to 9 dpi in seedlings of all treatments, with the highest activity recorded for this enzyme in the last measurement (21 dpi) (Figure 6e). Seedlings of the treatment INO + AZA showed the highest PPO activity at 21 dpi, followed by the INO, CONTROL, and INO + AZA + MEF treatments. Plants from these treatments did not differ statistically for this enzyme (Figure 6e). It is worth noting that since these treated plants had a similar PPO activity to that observed in the CONTROL treatment at 21 dpi, this might be an indication that PPO activity increased over time but did not vary much between treatments, regardless of AZA or MEF applications (Figure 6e).

## 4. Discussion

This study complements earlier findings on the effects of NeemAzal^®^-T/S (AZA), a neem-based botanical formulation, on sunflower downy mildew. Previous studies have demonstrated some inhibitory effects of AZA on *P. halstedii* sporangial germination under in vitro conditions (indicating some direct antifungal effect on the pathogen), and that its pre-treatment, rather than post-treatment, is more effective in controlling disease symptoms under in vivo conditions. Since the mechanisms which resulted in its induced systemic resistance (ISR) were still unclear, a further investigation into plant antioxidant responses involved in AZA-induced resistance was performed in our study, in order to clarify its physiological basis [7,9,13]. A direct effect on the pathogen promoted by AZA is suggested because it is commonly used as a biopesticide and may also act as a fungicide [7,9,13,36]. However, our results demonstrated that its primary protective role is also based in inducing host defence mechanisms prior to infection.

Pathogen infection often causes dwarfing in plants due to hormonal and metabolic changes. Even though *P. halstedii* infection has been associated with increased phenolic accumulation and IAA-oxidase activity, leading to degradation of indole-3-acetic acid (IAA) and inhibition of plant growth [37], treatments inducing resistance to downy mildew have also been shown to increase POX and PPO activities, which are involved in phenolic metabolism and can influence plant growth and development [22,23]. Given these insights, it is plausible to say that AZA-induced resistance involves the modulation of phenolic metabolism and hormonal pathways. However, the growth-promoting effects by AZA observed in our study can also be attributed to a to a significant enhancement in the activity of ROS-scavenging enzymes, thereby supporting an induced resistance mechanism rather than a solely direct antifungal effect at the studied AZA dose, which contributed to the observed plant growth responses [13,38].

Following pathogen recognition, ROS production represents one of the earliest plant defence responses [25]. In our study, infection by *P. halstedii* alone triggered H_2_O_2_ accumulation, confirming of oxidative stress responses, as reported in other studies [7,21,38,39]. Depending on the pathogen lifecycle, ROS is considered as the first line of defence against pathogen invasion and may act as a toxic and direct antimicrobial agent [18]. Therefore, H_2_O_2_ production in infected sunflower seedlings is an essential element of the disease resistance mechanism, which is involved directly or indirectly in restricting pathogen growth [16]. Nevertheless, under plant-pathogen interactions, the equilibrium between ROS production and scavenging is broken, and an excess in ROS production disrupts cellular homeostasis, leading to oxidative damage to proteins, lipids, and DNA [18,40]. In addition to that, MDA, a marker of lipid peroxidation, progressively increases in inoculated and untreated seedlings, particularly at later stages of infection [24,38,41].

Plants can protect themselves against oxidative stress by triggering the activation of various antioxidant enzymes. SOD, CAT, APX, POX and PPO are suggested to be associated with host defence mechanisms since these enzymes play a crucial role in ROS scavenging, protecting plant cells from oxidative damage produced in response to biotic and abiotic stresses [24,38,41,42]. In our study, inoculated and non-treated plants were unable to activate these enzymes effectively, leading to increased MDA accumulation. This was expected as the subsequent accumulation of H_2_O_2_ levels in biotrophic plant-pathogen interactions can lead to a hypersensitive response (HR), resulting in cell death to prevent pathogen invasion [18]. Our results revealed that both AZA and MEF reduced MDA levels, while the combination (AZA + MEF) also decreased H_2_O_2_ content, suggesting complementary roles with AZA inducing plant defenses and MEF directly limiting pathogen development. Moreover, the induction or suppression of ROS generation in susceptible and treated seedlings observed in this study can also be related to the activity of antioxidant enzymes which decreased or increased H_2_O_2_ levels. This could either be a direct result of H_2_O_2_ decomposition (a chemical reaction where H_2_O_2_ breaks down into water and oxygen) by CAT and APX; by its oxidation by POX (which catalyses the oxidation of various substrates using H_2_O_2_ as an electron acceptor); or its dismutation by SOD (with superoxide radicals being converted into oxygen and H_2_O_2_, which is then further processed by other enzymes [16]. Therefore, it is suggested that the observed H_2_O_2_ accumulation in inoculated and non-treated plants was not related to an increase in SOD activity, but rather due to pathogen infection. This could also be explained by the observed decrease in ROS (H_2_O_2_) scavenging peroxidase enzymes (CAT, APX and POX), which is particularly important in biotrophic plant-pathogen interactions, as in this case, it is known that the H_2_O_2_ generated by the pathogen itself or by SOD can act as a signalling molecule that regulates plant development, stress adaptation, and programmed cell death (PCD) responses [43].

Interestingly, inoculation with *P. halstedii* alone did not change SOD, CAT, APX, POX and PPO activities in sunflower seedlings at different stages of infection (3, 9 and 21 dpi) in our study. This indicates that without any kind of treatment, plants were not able to fight against pathogen attack and prevent or reduce oxidative stress. However, when applied alone or in combination, AZA increased CAT and APX activities, which were responsible for catalysing the conversion of H_2_O_2_ into H_2_O, specially APX. This can be evidenced by the fact that when APX activity decreased, H_2_O_2_ levels increased in both treatments (INO + AZA and INO + AZA + MEF), whereas the same pattern was not observed for any of the other peroxidases evaluated in this study. The ascorbate-glutathione cycle is a major H_2_O_2_ detoxifying system in plant cells. In this system, APX plays a key role catalysing the conversion of H_2_O_2_ into H_2_O, using ascorbate as a specific electron donor. In addition to that, the expression of APX genes is regulated in response to biotic and abiotic stresses as well as during plant development [44]. Even though H_2_O_2_ increased in seedlings from these treatments (INO + AZA and INO + AZA + MEF), MDA levels decreased to a much lower degree than that observed in non-treated and inoculated seedlings, not varying over time.

Peroxidases, including CAT, APX and POX play a pivotal role in scavenging and H_2_O_2_ detoxifying generated during pathogen invasion and other metabolic processes in plant cells [45]. Additionally, PPO is a key enzyme in plant defence mechanisms against pathogen invasion, primarily due to its involvement in the oxidation of polyphenols into quinones and its contribution to cell wall lignification [23]. At a later stage of infection (21 dpi), a significant increase in APX and POX activities was only observed in plants subjected to the combined treatment (INO + AZA + MEF), whereas PPO activity was elevated exclusively in the INO + AZA treatment. These results suggest that CAT activity was triggered early, but it does not play a major role in defense responses against *P. halstedii* infection through H_2_O_2_ detoxifying, since it progressively decreased over time. Moreover, the high PPO activity observed at 21 dpi may be associated with plant developmental stage, as older seedlings typically exhibit increased lignin deposition, contributing to structural reinforcement and enhanced defense.

Accordingly, the pronounced decrease in CAT and APX activities in inoculated and non-treated seedlings may have contributed to the development of disease symptoms (sporulation). On the contrary, the increase in the activity of major antioxidant enzymes in the INO + AZA and INO + AZA + MEF treatments could explain why disease incidence was reduced if compared to inoculated and non-treated plants (INO treatment). This can be explained by the fact that to maintain normal growth, these enzymes, especially APX, showed an active response against cell damage. This resulted in much lower MDA levels in plants which were treated with AZA or AZA + MEF, contributing to the maintenance of cell membrane integrity and improvement of the antioxidant activity of different enzymes. It also indicates some degree of resistance, which is in accordance with other studies on the use of different resistance inducers [22,23].

Finally, our results suggest that the high production of ROS (H_2_O_2_) and the capacity of treated plants to regulate its concentrations associated with low levels of MDA might contribute to increased resistance against downy mildew infection, since this resulted in much less oxidative damage in plants. ROS are involved in plant disease defence responses in different ways. They can reinforce plant cell wall through cross-linking reactions of lignin and proteins; can act as toxic agents either against the host plant cells through a HR and systemic acquired resistance (SAR), or against the pathogen itself by killing or stopping its growth; or by participating as secondary messengers in signalling routes leading to the activation of plant defence-related enzymes and genes [18,40,46]. Therefore, an increase in H_2_O_2_ levels, either by enhanced production or decreased ROS scavenging may contribute to induced-defence responses in susceptible sunflower seedlings against downy mildew via HR, since it is considered a biotrophic pathogen [18,47,48]. However, an imbalance between H_2_O_2_ generation and ROS scavenging enzymes may reflect either a defence mechanism by plants or a pathogenicity strategy by this oomycete [48,49]. When correlating our results for MDA and H_2_O_2_ content with the antioxidant enzymatic defense systems evaluated in this study, it was observed that only the treatment INO + AZA + MEF resulted in reduced H_2_O_2_ production and MDA levels. Therefore, the combined use of AZA + MEF can reduce MDA levels, contributing to the maintenance of membrane integrity and improvement of the antioxidant activity of different enzymes. This reduction might be related to the use of AZA acting as an inducer of many plant defence mechanisms, triggering antioxidant defence responses and stimulating the transcription of resistance-related genes, reducing disease severity and increasing the activity of antioxidant enzymes which can lead to the reduction of oxidative damages [14,50].

## 5. Conclusions

The mechanisms by which NeemAzal^®^-T/S (AZA) modulates plant physiology and biochemistry through the activation of antioxidant defence systems are not yet fully understood. Nevertheless, our results demonstrated that AZA can act as a resistance inducer by enhancing ROS-scavenging enzymes, while Mefenoxam (MEF) provides direct chemical suppression of the pathogen. The combined application of both treatments (AZA + MEF) reduced disease symptoms, decreased oxidative stress markers (MDA and H_2_O_2_), and promoted higher activities of APX, POX, and PPO at later infection stages. In addition to that, the combined treatment was also more effective in restoring plant height, even when MEF dose was halved, suggesting a possible synergism effect between AZA and MEF. However, in view of our results, there is a need to correlate our findings to a broader plant-defence response through the expression of resistance genes, to increase the understanding of enzymatic antioxidant defence mechanisms and how they are activated in sunflower seedlings inoculated with downy mildew.

## Data Availability

All data generated or analyzed during this study are included in the manuscript and its Supplementary Materials (if requested).

## Supplementary Materials

The following supporting information can be downloaded at: https://www.mdpi.com/article/10.3390/plants14223481/s1, Figure S1. Frequency of sporulation at 09 dpi in susceptible sunflower seedlings inoculated with downy mildew (pathotype 704) and treated with NeemAzal®-T/S (AZA), Mefenoxam (MEF) and AZA+MEF. From left to right, treatments were as following: CONTROL, INOCULATED, INO+AZA, INO+MEF and INO+AZA+MEF. Figure S2. Initial (9 dpi-top) and final (21 dpi-bottom) plant height in susceptible sunflower seedlings inoculated and non-inoculated with *P. halstedii* (pathotype 704) and treated with NeemAzal^®^-T/S (AZA), Mefenoxam (MEF) and AZA+MEF. From left to right, treatments were as following: CONTROL, INOCULATED, INO+AZA, INO+MEF and INO+AZA+MEF.
