# Supplementary material for: NeemAzal®-T/S Can Trigger Early Defense Responses in Susceptible Sunflower Seedlings Inoculated with Plasmopara halstedii: An Approach Based on the Enzymatic ROS Scavenging System
Source: Plants (Basel). 2025 Nov 14;14(22):3481. doi: 10.3390/plants14223481 (PMC12656035; doi:10.3390/plants14223481)
Supplement: Supplementary file 1 [file plants-14-03481-s001.zip › plants-3905514-supplementary.pdf]

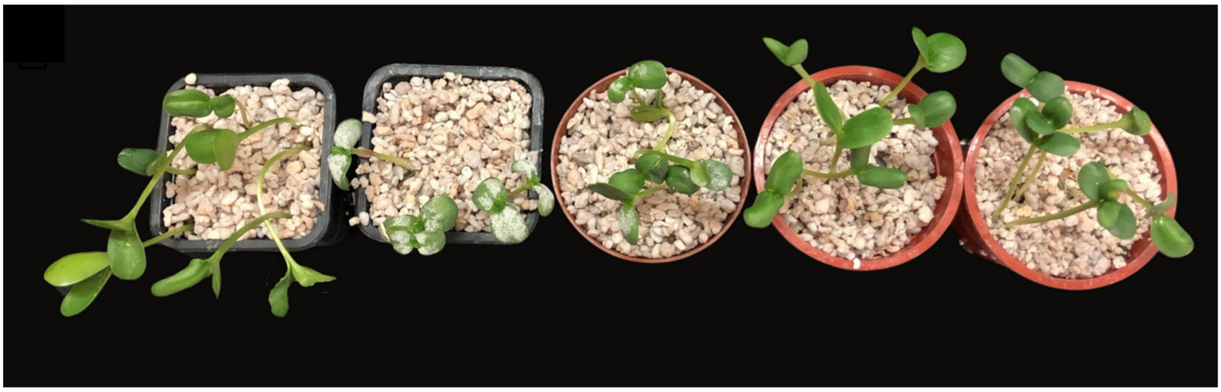

Supplementary Figure S1. Frequency of sporulation at 09 dpi in susceptible sunflower seedlings inoculated with downy mildew (pathotype 704) and treated with NeemAzal®-T/S (AZA), Mefenoxam (MEF) and AZA+MEF. From left to right, treatments were as following: CONTROL, INOCULATED, INO+AZA, INO+MEF and INO+AZA+MEF.

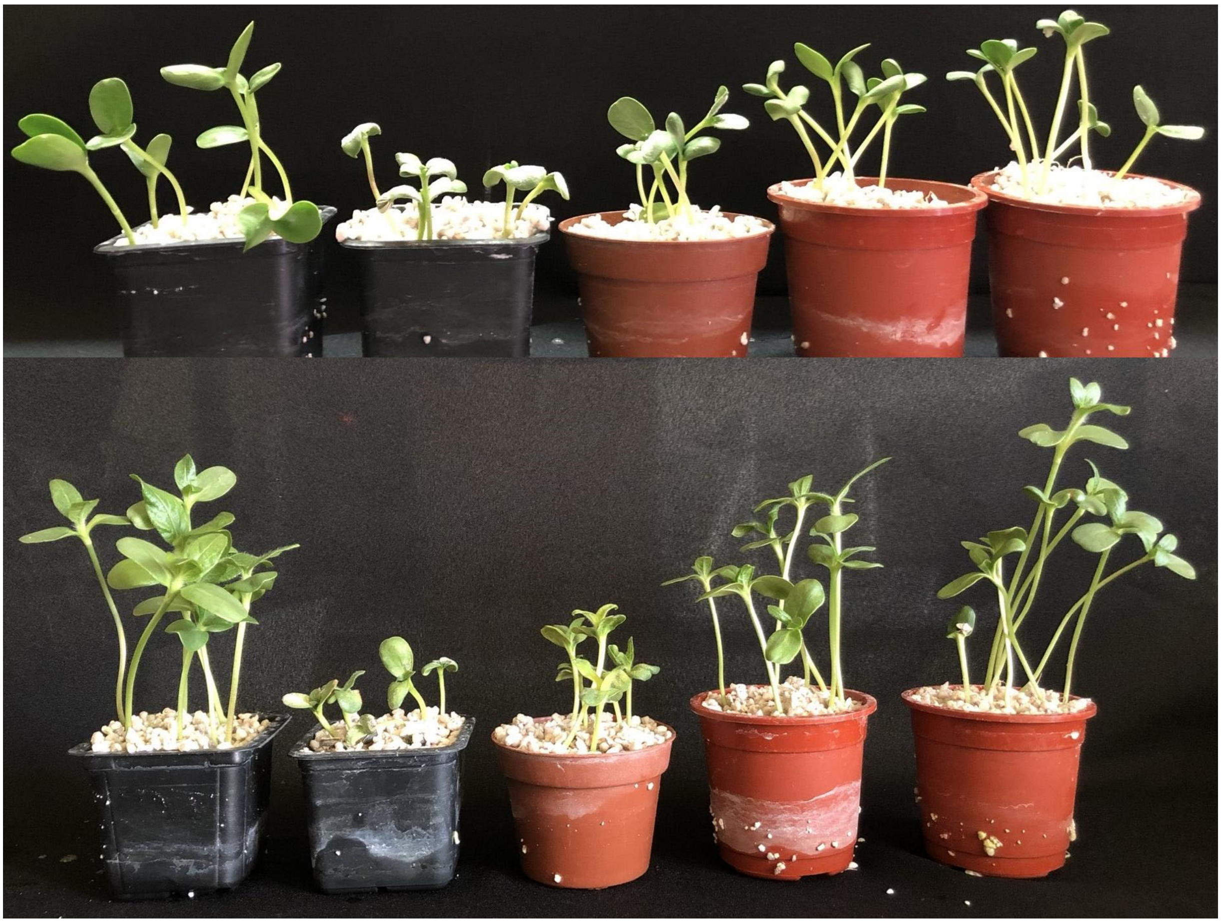

Supplementary Figure S2. Initial (9 dpi-top) and final (21 dpi-bottom) plant height in susceptible sunflower seedlings inoculated and non-inoculated with P. halstedii (pathotype 704) and treated with NeemAzal®-T/S (AZA), Mefenoxam (MEF) and AZA+MEF. From left to right, treatments were as following: CONTROL, INOCULATED, INO+AZA, INO+MEF and INO+AZA+MEF.
